# Primary biliary cholangitis first demonstrated in pregnancy: a case report

**DOI:** 10.1186/s13256-022-03260-7

**Published:** 2022-01-31

**Authors:** Daniela Melo, Ana Luísa Areia, Paulo Moura

**Affiliations:** 1 Obstetrics A Unit, Hospitais da Universidade de Coimbra, Centro Hospitalar e Universitário de Coimbra, Coimbra, Portugal; 2 Faculty of Medicine, University of Coimbra, Coimbra, Portugal

**Keywords:** Primary biliary cholangitis, Pruritus, Pregnancy, Case report

## Abstract

**Background:**

We present a case of primary biliary cholangitis diagnosed during pregnancy. Diagnosis of this entity in pregnancy is infrequent, and when everything seemed to point to a simple obstetric cholestasis, close attention to the details of the clinical history was required to raise suspicion of the true diagnosis.

**Case presentation:**

We present a 37-year-old Portuguese Caucasian patient who complained of generalized pruritus and showed alteration in hepatic function tests with a cholestatic pattern. The first diagnostic hypothesis was intrahepatic cholestasis of pregnancy, and she began treatment with ursodeoxycholic acid, which resulted in slight improvement of cholestasis. Her pregnancy was also complicated with occlusive hemorrhagic placenta, and at 30 weeks she underwent emergency cesarean section due to heavy blood loss. However, careful observation of clinical and laboratory findings, postpartum evolution, and a multidisciplinary approach to the patient led to the probable diagnosis of primary biliary cholangitis.

**Conclusions:**

Physiological changes during pregnancy can mimic chronic liver disease that can only be revealed at this stage, having an impact not only on the pregnancy but on the entire future of the woman.

## Background

Normal pregnancy involves numerous physiological, metabolic, and immunological changes that are necessary for the successful development of the fetus [1]. By increasing the metabolism and concentration of sex hormones and changes in the dynamics of plasmatic fluids (decreased percentage of cardiac output to the liver, decreased blood pressure and systemic vascular resistance), these changes may extend to the maternal liver, becoming apparent through signs and symptoms such as palmar erythema and telangiectasias (in 60% of pregnant women), pruritus, and changes in the hepatic biochemical profile, which may mimic chronic liver disease [2, 3].

Usually during pregnancy, levels of alanine aminotransferase, aspartate aminotransferase, and γ-glutamyl transferase are within normal or slightly decreased values due to hemodilution. Total and indirect bilirubin may be slightly decreased throughout pregnancy, and direct bilirubin decreases during the second and third trimesters. On the other hand, due to the increased production of its placental and bone isoenzyme, alkaline phosphatase increases markedly (2–4 times) during the third trimester of gestation. The total concentration of bile acids does not change, and albumin levels decrease 20–40% due to the increase in plasma volume and the decrease in its production. Conversely, production of cholesterol and triglycerides increases. There is no change in activated prothrombin or in partial thromboplastin times, despite the increased hepatic production of factors V, VII, and VIII and fibrinogen [3, 4]. The incidence of these changes is approximately 3–5% [3].

Nevertheless, the possibility of underlying liver disease unmasked by pregnancy should be considered since it may have different prognostic implications for both mother and fetus. Thus, when cholestasis appears in pregnancy, this does not always imply the diagnosis of intrahepatic cholestasis of pregnancy (ICP), the most frequent cause of cholestasis in pregnancy [5].

Liver diseases that occur in pregnancy may be divided into three groups: those exclusive to pregnancy, others discovered or exacerbated during pregnancy, and some that concur with pregnancy. The combination of clinical history, physical examination, careful interpretation of liver function tests, viral infection markers, autoimmunity study, and abdominal ultrasonography are extremely important for diagnosis [2, 3, 5]. Sepsis as well as radiation can worsen the prognosis of autoimmune diseases such as primary biliary cholangitis [6–8].

## Case presentation

A 37-year-old Portuguese Caucasian patient began to complain of generalized pruritus at 12 weeks’ gestation, predominantly in the palms of the hands and soles of the feet, with progressive worsening and alteration of hepatic tests with a cholestatic pattern (Table 1) but no other associated symptoms. Physical examination showed lesions of grattage throughout the body, normal blood pressure, normal neurological examination, absence of edema, and on abdominal palpation, spleen and liver not palpable. The patient underwent abdominal ultrasound that showed a homogeneous hepatic structure with a well-delimited echogenic solid nodule, in segment VII, suggestive of hemangioma (0.8 cm), without any other alterations; and splenomegaly of regular contour.

**Table 1:** Initial study

Normal	Abnormal	Normal range
Hemogram	AST 90 U/L	<31 U/L
Prothrombin time and activated partial thromboplastin time	ALT 170 U/L	<34 U/L
	ALP 848 U/L	30–120 U/L
	γ-GT 86 U/L	<38 U/L
	LDH 166 U/L	<247 U/L
	Bile acids 25.7 mmol/L	<6.0 mmol/L
	Total bilirubin 1.0 mg/dL	0.3–1.2 mg/dL

*AST* aspartate aminotransferase, *ALT* alanine aminotransferase; *ALP* alkaline phosphatase, *γ-GT* γ-glutamyl transferase, *LDH* lactate dehydrogenase

This was her second pregnancy. The first one, in 2016, ended with an eutocic delivery of a 2665-g child, induced at 37 weeks by oligohydramnios. The current pregnancy had been monitored since the first trimester under a private regimen. At the onset of signs and symptoms of cholestasis, she was then followed by the Nephrology/Obstetrics Unit and by Internal Medicine Unit, both in the University Hospital Center of Coimbra.

**Fig. 1:**
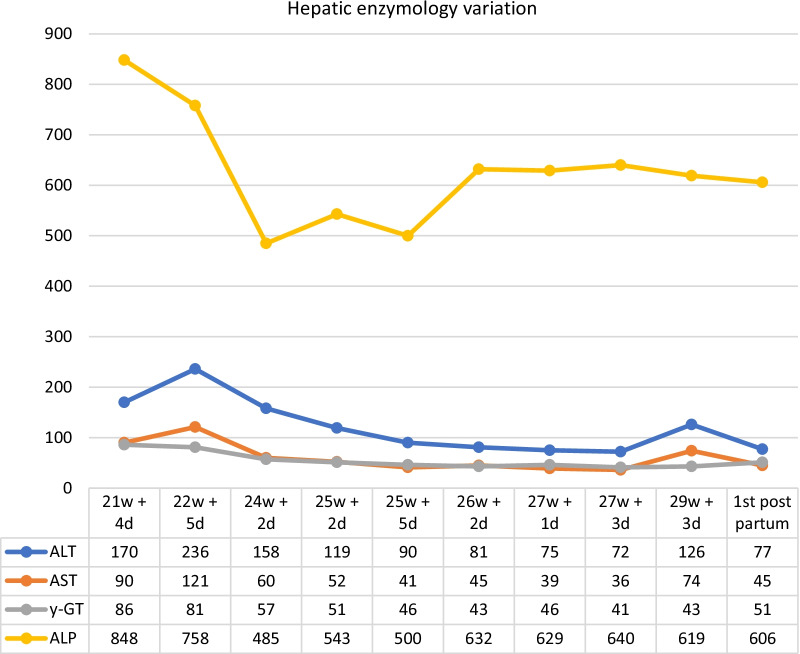
Hepatic enzymology variation throughout pregnancy (U/L)

**Fig. 2:**
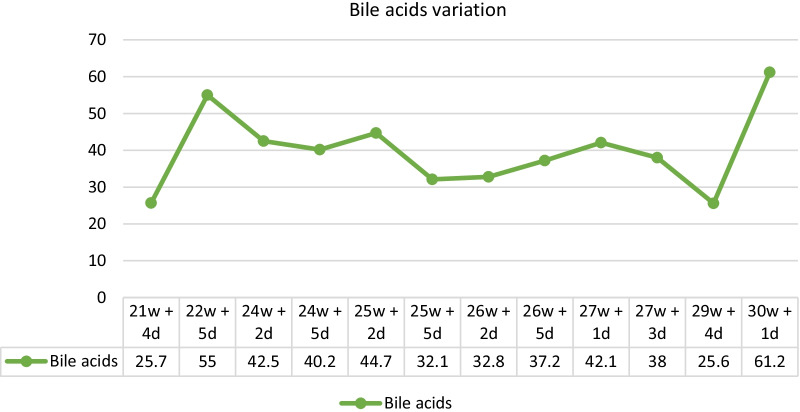
Bile acids variation throughout pregnancy (mmol/L)

Due to the symptoms and the alterations in hepatic laboratory tests, the first presumed hypothesis was intrahepatic cholestasis of pregnancy. Therefore, treatment with ursodeoxycholic acid 250 mg twice daily was initiated. However, to better clarify and exclude other differential diagnoses, a complete analytical study was performed, including virology, autoimmunity, urine examination with protein/creatinine ratio (Table 2), and liver ultrasound. In addition to bile acids of 55.3 mmol/L, the patient presented worsening liver function tests, maintaining a cholestatic pattern. There was a good response to ursodeoxycolic acid, with improved cytolysis and slight improvement of cholestasis (Figs. 1 and 2). Doppler hepatic ultrasound revealed hepatic portal vein with normal velocities (0.2 m/s), hepatopetal arteriopathic with normal resistance indexes (0.6 m/s), and modulated hepatobiliary hepatic veins. Considering the clinical and laboratory findings, primary biliary cholangitis became one of the most probable diagnoses. However, it was not possible to rule out the possibility of intrahepatic cholestasis of pregnancy, and as the clinical course did not change with the new diagnostic hypothesis, we maintained therapy with ursodeoxycholic acid (but increasing the dose to 500 mg twice daily) and regular maternal–fetal surveillance, with periodic reassessment of serum bile acid and fetal Doppler.

**Table 2:** Analytical study

**Positive exams**	**Negative exams**
Anti-nuclear (ANAs) antibodies with centromeric pattern	Hepatitis A, B, C e E
Anti-M2 antibody (pyruvate dehydrogenase)	Cytomegalovirus
Immunoglobulin M	Immunoglobulin A
	Alpha 1 antitrypsin
	Ceruloplasmin
	Protein/creatinine ratio

In addition, there were two hospitalizations due to low occlusive hemorrhagic placenta (at 24 weeks and at 29 weeks). Accordingly, fetal pulmonary maturation was performed with dexamethasone. She maintained regular fetal and analytic surveillance during hospitalization. At 30 weeks 2 days, emergency cesarean section was performed due to heavy blood loss and analytical aggravation, reaching serum levels of bile acids of 61.2 mmol/L. A boy was born, with 1430 g and Apgar score 8/9/9, being admitted to the Newborn Intensive Care Unit.

At 6 weeks postpartum, she maintained pruritus, especially on the palms of her hands and the soles of her feet, especially at night; abnormal liver tests prevailed and a fasting glucose anomaly appeared (hemogram, prothrombin time, and activated partial thromboplastin time without changes, fasting glucose 115 mg/dL, AST 129 U/L, ALT 199 U/L, ALP 672 U/L, γ-GT 178 U/L, LDH 177 U/L). She underwent abdominal magnetic resonance imaging (MRI) with contrast, which showed an enlarged liver at the expense of right lobe hypertrophy with regular contours and a globally homogeneous signal, two nodular formations in segment VII compatible with hemangiomas, and absence of intra- and extrahepatic biliary dilatation.

Therefore, given the clinical and analytical evolution in the postpartum period, the definitive diagnosis was primary biliary cholangitis, with first manifestation in pregnancy.

## Discussion and conclusion

In this clinical case, the first diagnostic hypothesis was ICP, also known as obstetric cholestasis. In fact, this is the most common liver disease of pregnancy (with prevalence of 0.1–1.5% of all pregnancies in Europe), the most frequent cause of cholestasis in pregnancy, and the second major cause of jaundice in pregnancy, shortly after viral hepatitis [3, 5, 9, 10]. Still, it manifests itself mainly in the second and third trimester of pregnancy and resolves with childbirth [1, 3–5, 9, 10].

In our case, as a diagnosis of exclusion, the fact that cholestasis occurred early in pregnancy, the lack of history of cholestasis in the previous pregnancy or family history (which occurs in 30% of cases of ICP), and persistence of signs and symptoms after childbirth led to the definitive diagnosis of primary biliary cholangitis (PBC).

PBC is an autoimmune liver disease that represents one of the most important chronic cholestatic diseases [1, 2, 5, 11–14]. It is characterized by progressive destruction of small bile ducts mediated by T cells, which may lead to cirrhosis and considerable morbidity and mortality [2, 5, 11, 13, 15]. In 2014, the nomenclature changed from “primary biliary cirrhosis” to “primary biliary cholangitis” to better describe the natural history of the disease. In epidemiological terms, countries in Northern Europe and America show higher frequency of the disease, where females are the most affected (ratio 9–22:1) [2, 5, 11–15]. Typically, it affects postmenopausal women, but 25% are already diagnosed during the fertile period due to the recent improvement of diagnostic tests [1, 2, 5, 11, 12, 14]. The risk factors associated with PBC are family history, presence of another autoimmune disease, history of vaginal and urinary infections, smoking habit, and exposure to nail varnish and hair dye [2, 5, 13]. The use of the contraceptive pill seems to be a protective factor for the development of the disease [13].

Most patients are asymptomatic at diagnosis; however, pruritus and, more often, fatigue (in approximately 75%) may be present. PBC may lead to lipid changes and be associated with extrahepatic autoimmune events (in more than 80%), although these findings are nonspecific [2, 5, 11, 13].

Onset of PBC during pregnancy may be a consequence of drastic changes in the hormonal profile and the response of the immune system [12, 15]. In fact, to avoid immune rejection of the fetus, there is a shift in the TH1 cell response to a TH2 humoral response, potentiated by the large increase in steroid hormone levels during gestation. The diagnosis in pregnancy should be considered when the pruritus begins at precocious gestational age, based on the combination of a high TGP value in the absence of extrahepatic obstruction (at least 1.5 times the normal value for at least 6 months) and in the presence of antimitochondrial antibody (positive in 90 to 95% of cases with specificity of 98%) [2, 5, 11, 13]. There may also be marked γ-GT elevation and a greater increase than may be expected from ALP [13]. Antinuclear antibodies are positive in about 35% of cases; in 90% of cases, there is hypergammaglobulinemia, mainly at the expense of immunoglobulin M; and centromeric autoantibodies are present in 10–15% of cases [2, 5, 13]. Hepatic biopsy, although not necessary, may help in doubtful cases and may evaluate disease activity [11, 13].

Follow-up of the pregnant woman with PBC should be multidisciplinary and include maternal and fetal, clinical and ultrasound monitoring [2, 13].

Ursodeoxycholic acid (UDA) is the most studied and most recommended treatment, although its use is not approved by the US Food and Drug Administration (FDA) during pregnancy (category B). Its administration has proved to be effective in pruritus treatment, improvement of biochemical profile, delayed histological progression, and disease control; on the contrary, it does not appear to have maternal or fetal risks [3, 11, 13, 14]. Adverse effects at therapeutic dosages (13–15 mg/kg/day) are rare and moderate, mainly idiosyncratic [11]. When improvement of pruritus is insufficient, the dosage of UDA can be increased to 20–25 mg/kg/day. Cholestyramine (4–12 g/day) or rifampicin (150–300 mg twice daily) may be an option as they capture bile acids and prevent their reabsorption. Also, antihistamines, due to their sedative characteristics, may be antipruritic [13].

As in ICP, the most recent literature recommends terminating pregnancy at 37–38 weeks, to minimize the risk of fetal death in utero. In literature, there are no recommendations regarding mode of delivery, so the multidisciplinary approach seems to be the most appropriate for decision-making [13]. In this clinical case, the decisive route of delivery was urgent cesarean section, due to heavy blood loss.

It is known that pregnancy may affect autoimmune diseases, but vice versa, pregnancy may also be affected by them. Thus, pregnancy is considered to be at high risk [15] in most autoimmune diseases. However, several studies have shown that the outcome of autoimmune diseases is highly variable during pregnancy [14].

There is little information and consensus in literature about obstetric outcomes in pregnant women with PBC, as well as on the effect of pregnancy in the course of the disease [1, 3, 14, 15]. Nevertheless, the most common opinion with the greatest consensus is clinical remission or stabilization of the disease, with a decrease in antimitochondrial antibodies titers, suggesting that pregnancy results in immunological tolerance in these patients [1, 2, 11, 14, 16]. On the other hand, the course of pruritus is unpredictable [2, 11, 14]. The problem most reported is occurrence of placenta previa (1% of cases) [2, 15], which we also found in this clinical case. After delivery, 60% of women show exacerbation of the disease, with antimitochondrial antibody titles returning to their basal levels [2, 13, 14]. In terms of fetal outcomes, studies have shown that teh serum concentration of bile acids is relevant regarding the risk of preterm delivery, anoxia, and fetal death [1]. However, overall, outcomes appear to be good when the disease is well controlled.

Thus, it is important to diagnose liver disease during pregnancy, since early diagnosis can improve maternal and fetal outcomes and decrease morbidity and mortality. Finally, following the presumptive diagnosis of cholestasis in pregnancy, the persistence of a biological cholestasis, exceeding 3 weeks postpartum, prompted the diagnosis of chronic cholestasis, and in particular of PBC, with first manifestation in pregnancy.

## Data Availability

All data generated or analyzed during this study are included in this published article.
